# The Diseases of the Male Urethra

**Published:** 1899-03

**Authors:** 


**The Diseases of the Male Urethra.** By R. W. Stewart, M.D. Pp. viii., 221. New York: William Wood and Company. 1896.

This is a very useful book, which treats of inflammatory affections of the urethra and their result in the form of stricture. As gonorrhœa is so common it is worth while devoting some considerable time to the study of this disease and its sequelæ. Modern methods of diagnosis, such as the use of the endoscope, are fully described, and the affections of different portions of the urethra considered in separate chapters. The inflammatory diseases of the seminal glands which are connected with the urethra are also considered. We were rather staggered by the word "Cowperitis," which, although not new, is one against which we must enter an etymological protest. There are some good pictures of the anatomy of the urethra and useful representations of instruments employed in urethra diagnosis and treatment, and the book is a decided acquisition to the literature of the subject. The fact that the publishers' work has been done in so excellent a fashion makes it all the more acceptable.

